# Early detection of intractable postpartum hemorrhage

**DOI:** 10.1038/s41598-025-96114-3

**Published:** 2025-04-03

**Authors:** Saori Yoshimura, Yutaka Iwagoi, Fumitaka Saito, Chisato Kodera, Rumi Sasaki, Munekage Yamaguchi, Takashi Ohba, Eiji Kondoh

**Affiliations:** Department of Obstetrics and Gynecology, Faculty of Life Sciences, Kumamoto University, 1-1-1, Honjo, Chuo-Ku, Kumamoto City, Kumamoto 860-8556 Japan

**Keywords:** Clot evacuation, Early diagnosis, Early stratification, PPH, PRACE simulation, TI-V, Medical research, Risk factors, Signs and symptoms

## Abstract

Postpartum hemorrhage (PPH) is a leading cause of maternal mortality worldwide, with timely detection complicated by the inability to visualize bleeding within the uterine cavity. This study aimed to develop a method for early detection of intractable PPH, characterized by arterial contrast extravasation on dynamic CT (PRACE), often requiring uterine arterial embolization. The study comprised two components: (1) an ex vivo study, evaluating the PRACE visualization model using a postpartum uterine cavity simulation, and (2) an in vivo study assessing whether the time interval for bleeding to appear at the vagina (TI-V) could serve as an indicator of intractable PPH. The ex vivo simulation of PPH at various flow rates suggested that the overflow time from the uterine cavity may assist in stratifying life-threatening PPH. In the in vivo study, TI-V was measured in cases of uncomplicated vaginal delivery and severe PPH, including those transported for treatment. The results indicated that a TI-V of less than 2 s strongly predicted intractable PPH, with a positive predictive value of 100% and a negative predictive value of 98.2%. This straightforward method could significantly improve maternal health outcomes by enabling early identification and management of severe PPH.

## Introduction

Postpartum hemorrhage (PPH) remains a leading cause of maternal mortality globally^1^, presenting a critical challenge to maternal healthcare. Although PPH has traditionally been defined based on blood volume loss (≥ 500 ml for vaginal delivery and ≥ 1000 ml for cesarean section), recent guidelines have evolved to emphasize clinical signs along with blood loss^2^. For instance, ACOG defines PPH as blood loss ≥ 1000 ml or any blood loss accompanied by signs of hypovolemia within 24 h after birth, regardless of delivery mode^2^. However, accurate blood loss estimation remains challenging in clinical practice, and the focus on volume-based definitions may delay recognition of rapidly developing, life-threatening PPH. Effective and timely management of PPH is essential^2–5^, as delayed response significantly increases mortality risk. Unlike in general surgical procedures, the confirmation of hemostasis in PPH is hindered by the inability to directly visualize bleeding points within the uterine lumen. Consequently, current hemostasis management protocols often fall short due to the lack of a clear definition of hemostatic failure.

To optimize PPH management, early recognition indicators of life-threatening maternal bleeding are crucial^2–5^. Our previous research introduced the concept of PPH resistant to treatment showing arterial contrast extravasation on dynamic CT (PRACE), characterized by arterial bleeding from a focal point within the uterine cavity, frequently necessitating uterine arterial embolization^6^. We hypothesized that visualizing PRACE using our previously developed silicone postpartum uterine cavity model^7^ might provide an effective indicator for stratifying intractable and life-threatening PPH.

This study aimed to: (1) evaluate the PRACE visualization model in vitro using a postpartum uterine cavity model^7^, and (2) assess in vivo whether the time interval for bleeding to appear at the vagina through external os of the uterus (TI-V), measured through three-step process, could serve as a valid indicator of intractable PPH. The novel, simple method for early detection of PPH proposed in this study has significant potential to improve maternal health outcomes worldwide.

## Methods

### Study I: ex vivo study: visualization of PPH

#### Simulated blood with glycerol solution

To create simulated blood with an outflow rate similar to that of real blood, glycerol solutions at concentrations of 40%, 50%, 60%, 70%, 80%, and 90% were prepared by adding saline to glycerol. A volume of 0.2 ml of either blood or one of the glycerol solutions was added to the rim of a flat plastic container (1-3145-03, 200 × 140 × 25 mm, AS ONE Corporation, Osaka, Japan). The container was then tilted at an angle of 37 degrees at room temperature to measure the time required for the liquid to flow from one end to the other. This angle corresponds to the slope between the anterior surface of the first sacrum and the coccyx—specifically, the angle at which hemorrhage flows from the uterine cavity to the dorsal surface of the upper birth canal relative to the horizontal plane—in the CT images used to construct the uterine cavity model^6^. Blood samples were obtained from three male and four non-pregnant female controls and used in the experiment. Each experiment was repeated three times.

#### Examination of CT images in PPH patients

Dynamic CT images of patients transported to the hospital between June 2020 and June 2021 for the management of severe primary PPH, occurring within the first 24 h after delivery, were reviewed. The analysis focused on the position of the vagina in the supine position. Additionally, the CT images were utilized to assess how blood pooled in the birth canal and to measure the distance between the most dorsal side of the vaginal canal and the posterior fornix of the vagina.

#### Simulation of PPH using a transparent uterine cavity model

A previously developed silicone postpartum uterine cavity model^7^ was placed with reference to the images to match the position of the uterus in the supine position. Dynamic CT analysis indicates that approximately half of intractable PPH cases exhibit contrast leakage in the early phase from the upper and lower uterine boundaries^8^. Therefore, a 23 G needle was inserted at the upper and lower boundaries of a silicone postpartum uterine cavity model, and an 80% glycerol solution colored with red food coloring agents was infused at rates of 200, 300, 350, 400, and 500 ml/h (Video S1). The simulated blood flowed through the uterine cavity, pooled in the lower part of the uterus, and eventually overflowed from the uterine model. The time interval of simulated blood overflow from the uterine model was measured. The same experiment was also conducted with a 23 G needle inserted into the anterior wall of the upper uterine lumen (Video S1), as approximately half of intractable PPH cases originate from the upper part of the uterus^8^. All experiments were performed in triplicate, each being repeated three times independently. The experimental video data are placed in an optional access repository and made available to interested parties.

### Study II: in vivo study: exploring indicators of intractable PPH

**Fig. 1:**
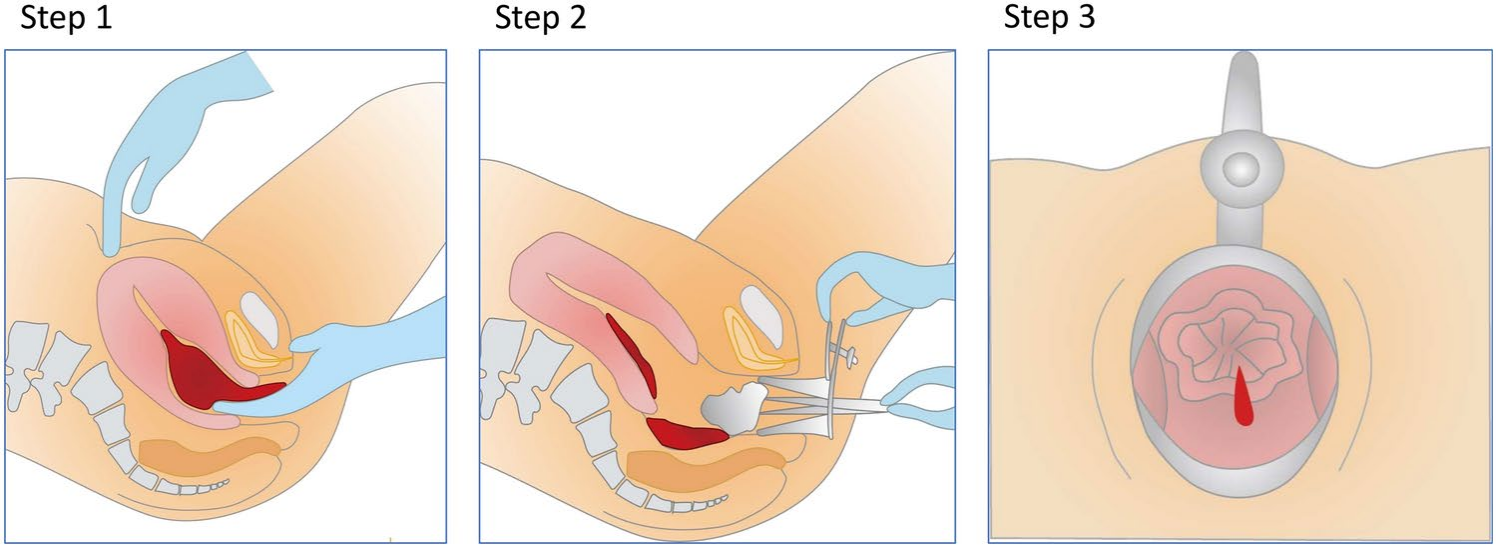
Three-step procedure for early detection of intractable postpartum hemorrhage. Step 1 (left): Caudal compression of the uterine fundus to expel clots from the birth canal using fingers. Step 2 (middle): wiping vaginal blood with gauze following inspection with a Cusco vaginal speculum. Step 3 (right): immediate measurement of the time interval for bleeding to appear at the vagina through external os after Step2.

Based on ex vivo experiments in the PPH model of Study I, it was hypothesized that the time taken for blood to flow from the uterine lumen into the vagina could reflect the severity of PPH. To test this hypothesis, the TI-V was prospectively measured in women who delivered vaginally at our institution between October 2022 and August 2023, and in those with severe PPH transported to our hospital after vaginal or cesarean delivery between March 2022 and May 2024. The measurement process involved: first, applying caudal compression to the uterine fundus to expel clots from the vaginal and lower uterine lumen using fingers (Step 1); second, wiping the vaginal blood with gauze following inspection with a Cusco vaginal speculum (Step 2); and third, immediately measuring the TI-V after Step2 (Step 3) (Fig. 1). The TI-V was measured upon completion of procedures such as suturing vaginal lacerations in vaginal delivery cases, or during the initial pelvic examination in transported cases. Exclusion criteria included hematomas in the vaginal wall, vulva, or perineal area, uterine inversion, uterine rupture, cervical laceration, and intraperitoneal bleeding. Cases in which the TI-V was not recorded in the medical record were also excluded from the analysis.

Medical records were reviewed to collect data on transported cases, including age, pregnancy history, gestational weeks at delivery, mode of delivery, amount of blood loss, transfusion volume, utilization of dynamic CT scan, cause of PPH, presence of PRACE, and hemostatic interventions. For cases in which no blood was seen at the vagina after more than 15 s, the TI-V was recorded as 15 s.

### Ethical considerations

Approval for the study protocol was obtained from the Institutional Review Board of Kumamoto University (No. 2632). All methods were carried out in accordance with relevant guidelines and regulations. For the ex vivo study, written informed consent was obtained from all participants from whom blood samples were collected. For the retrospective analysis of patient data in the in vivo study, an opt-out method was used to obtain consent, as approved by the Institutional Review Board.

### Statistical analysis

The flow rate of simulated blood into the transparent uterine cavity and the time interval of simulated blood overflow from the cavity model were assessed using linear regression analysis. Receiver operating characteristic (ROC) analysis was conducted using the TI-V to identify the optimal cutoff point for early detection of intractable PPH. Intractable PPH was defined as cases where patients required uterine artery embolization or bimanual compression-supported balloon tamponade^5^. Statistical analyses were performed using GraphPad Prism 10.2 (GraphPad Software, La Jolla, CA).

## Results

### Study I: ex vivo study

#### Simulated blood

The time taken for the glycerol solution to flow over the plastic container increased with concentration, from 1.6 ± 0.2 s for the 40% concentration to 9.0 ± 0.7 s for the 90% concentration (Figure S1). In experiments with blood (n = 7), the median flow time was 4.0 s [3.1–7.4], closely matching the 80% glycerol solution flow time (Figure S1). Therefore, 80% glycerol solution was used as the simulated blood in subsequent experiments.

#### Hematoma accumulation in the birth canal and position of the posterior vaginal wall

**Fig. 2:**
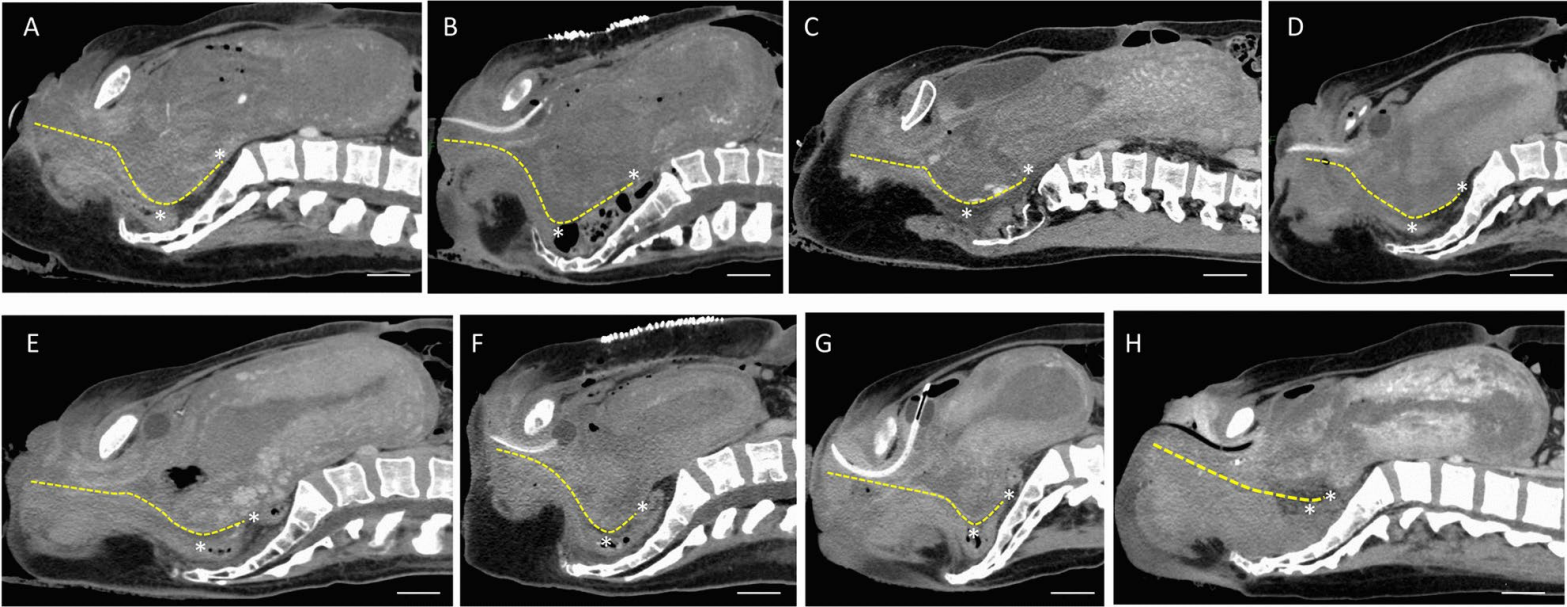
Displacement of posterior vaginal wall by clot accumulation in the birth canal. Sagittal images from the late phase of dynamic CT scans in patients transported for the management of severe primary postpartum hemorrhage (PPH) (**A**–**H**). Cases A and C showed contrast extravasation, with Case A involving a rupture of the lower uterine segment and Cases C and H resulting from vaginal lacerations. The remaining cases were due to uterine atony. The posterior vaginal walls are indicated by yellow dashed lines, and the asterisk marks the most dorsal aspect of the vaginal canal and the posterior fornix. The posterior vaginal wall was displaced dorsally due to the accumulation of blood clots in the birth canal (**A**–**G**). The distances between the asterisks, which may indicate the degree of upper vaginal extension, were as follows: A: 7.0 cm, B: 7.0 cm, C: 4.7 cm, D: 4.2 cm, E: 3.7 cm, F: 3.3 cm, G: 3.3 cm, and H: 0.9 cm. The scale bar indicates 4.0 cm.

During the study period, triple-phase dynamic CT imaging was performed on 8 cases of primary PPH (Fig. 2). The posterior vaginal wall was displaced dorsally due to the weight of the accumulated blood clots in the birth canal (Fig. 2A–G). Additionally, the posterior vaginal wall was extended cephalad as the uterine body was pushed upward by the clots. The distance between the most dorsal aspect of the vaginal canal and the posterior fornix of the vagina extended up to 7 cm (Fig. 2A, B). In contrast, minimal clot accumulation resulted in little to no dorsal or cephalad displacement of the posterior vaginal wall (0.2 cm, Fig. 2H). These findings suggest that the upper part of the birth canal may mask the actual degree of hemorrhage due to its capacity to retain a substantial amount of blood. Contrast extravasation was observed in 2 out of 8 cases in the early phase (Fig. 2A, C). Even in cases without contrast extravasation, moderate to high accumulation of clots in the birth canal was noted (Fig. 2B, D, F).

#### Simulated bleeding rate and time interval of overflow

**Fig. 3:**
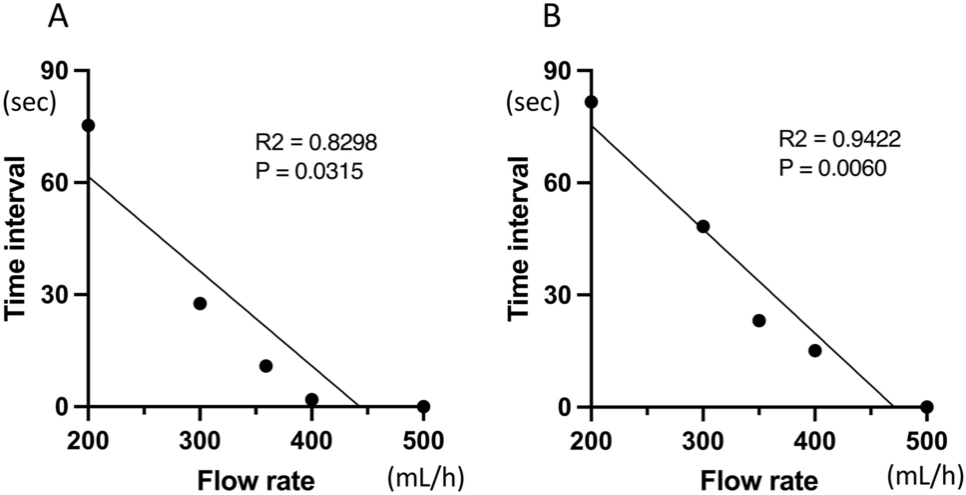
Bleeding rate and time interval of outflow in PRACE visualization model. Correlation of bleeding flow rate with the time interval of simulated blood overflow in the (**A**) lower and (**B**) upper uterus. PRACE; postpartum hemorrhage resistant to treatment showing arterial contrast extravasation on dynamic computed tomography.

In the PRACE model, assuming the bleeding point at the boundary between the upper and lower uterus, an increased drip flow rate resulted in a shorter time interval of overflow from the uterine model (R^2^ = 0.8298, *P* = 0.0315; Fig. 3A). Similarly, in the PRACE model with a bleeding point in the upper uterus, an increased simulated bleeding rate also resulted in a shorter time interval (R^2^ = 0.9422, *P* = 0.0060; Fig. 3B). At flow rates of 200–400 mL/h, the former model with the bleeding point closer to the uterine outlet showed a shorter time interval compared to the model with the bleeding point in the upper uterus (Fig. 3). At a flow rate of 500 mL/h, both models exhibited immediate overflow from the uterine lumen after flow initiation.

### Study II: in vivo study

**Fig. 4:**
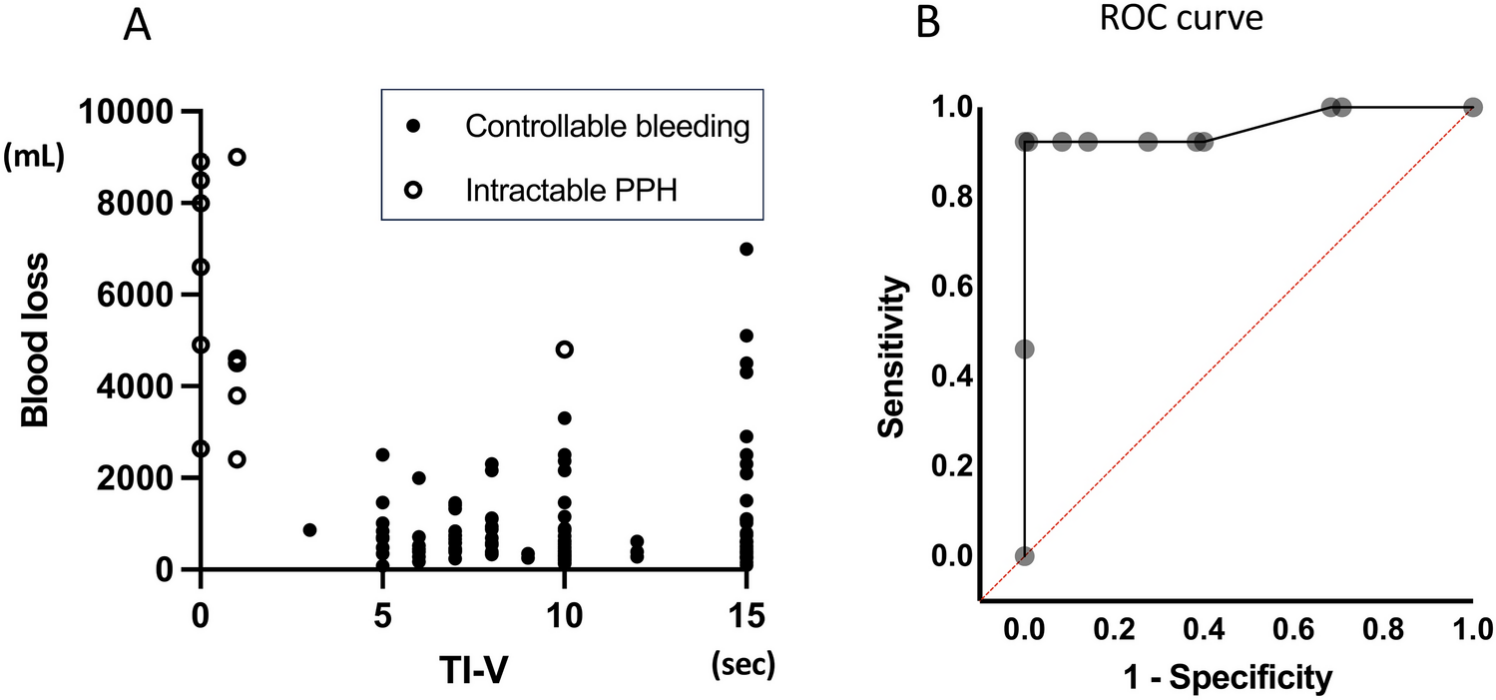
Time interval from clot expulsion to onset of vaginal bleeding. (**A**) Relationship between the time interval and blood loss volume. The white circle represents cases of PPH that were resistant to conventional hemostasis, while the black circle indicates cases that were successfully managed using standard hemostatic approaches. (**B**) ROC curve for predicting intractable PPH based on the TI-V. PPH, postpartum hemorrhage; TI-V, time interval for bleeding to appear at the vagina; ROC, receiver operating characteristic curve.

A total of 125 women delivered vaginally at our hospital between October 2022 and August 2023, of whom 106 were included in the analysis (data not shown). One case required uterine arterial embolization, with a TI-V of 0 s. Additionally, 42 women with severe PPH were transported to our hospital between March 2022 and May 2024, and 27 of these women were included in the analysis (Table S1). Eleven cases had a TI-V of 0 or 1 s. Dynamic CT scan was performed in 16 cases to guide treatment strategy prior to initiating new hemostatic techniques. Uterine artery embolization was required in seven cases, and bimanual compression-supported balloon tamponade^5^ was performed in ten cases with a diagnosis or suspicion of lower PRACE; five of these cases underwent both interventions. No cases required hysterectomy. The median TI-V, measured using the three-step maneuver, was 10 [0–15] s across 133 cases, including both uncomplicated deliveries and transported PPH cases (Fig. 4A). Twelve patients were classified as having intractable PPH, where conventional PPH management methods failed to achieve hemostasis. ROC analysis demonstrated that TI-V measured by the three-step process could predict intractable PPH, with an AUC of 0.8998 (Fig. 4B). A cut-off value of < 2 s yielded a sensitivity of 92.3%, specificity of 100%, a positive predictive value of 100%, and a negative predictive value of 99.2%.

## Discussion

The findings from this study provide valuable insights into the early detection and management of intractable PPH, a critical challenge in maternal healthcare. A straightforward three-step process for assessing the severity of PPH, which does not require special equipment, has the potential to identify PPH unresponsive to standard management at an early stage.

Managing PPH is essential for obstetricians to ensure maternal safety. Current PPH management follows an established stepwise protocol that begins with first-line interventions including uterine massage, administration of uterotonic agents, and tranexamic acid^2–6^. If bleeding persists, second-line interventions such as intrauterine balloon tamponade are employed^2,6^. Refractory cases necessitate invasive interventions, including uterine artery embolization or hysterectomy^2,6^. Despite these established protocols, the inability to directly visualize active PPH continues to pose a significant challenge in implementing timely and appropriate interventions. This limitation has historically hindered the advancement of innovative management strategies in this field, particularly in determining when to escalate from conservative to more aggressive treatments. By analyzing CT images of PPH and conducting PRACE simulations using a silicone-based postpartum uterine cavity model, we highlighted two important, yet often overlooked, aspects of PPH. First, the birth canal, particularly the upper vagina, can expand due to blood clots, allowing it to store a significant amount of blood. This expansion complicates the evaluation of PPH severity and can lead to an underestimation of blood loss. Of clinical importance, our analysis confirmed that the presence of blood clots does not necessarily indicate active bleeding, suggesting that high blood loss does not always correspond to life-threatening, intractable PPH. Second, the flow rate of blood per unit time determines the time required for the blood to overflow from cavity of the uterus. The findings from these ex vivo studies suggest that, rather than measuring the total blood loss, assessing the time required for accumulated blood in the uterine cavity to flow out to the vagina could facilitate early stratification of PPH severity. This PPH model may serve as a valuable tool for studying the dynamics of PPH and for disseminating new PPH management practices.

More than half of maternal deaths due to PPH are believed to be preventable, affecting not only low-income countries but also developed nations^9,10^. Poor outcomes following PPH are often due to delays in the identification and treatment of PPH^9–11^. Traditionally, PPH has been primarily assessed based on the amount of blood loss^10,11^. Current methods for evaluating blood loss during childbirth include visual estimation, the use of blood-collection drapes, and various other techniques^3,5,10,11^. However, a universally implemented and reliable method for measuring blood loss that improves maternal outcomes has not been fully established^4,10,11^. Commonly used methods for assessing blood loss typically require a significant amount of time, such as 30 min to an hour. In contrast, measuring the TI-V after performing a manual uterine massage to expel clots can be completed in less than one minute and can be implemented globally. Although clot expulsion has been reported as a simple clinical maneuver that may reduce the need for surgical intervention in the management of severe PPH^12^, no studies have attempted to stratify PPH by measuring TI-V. The TI-V was found to be a significant predictor of intractable PPH, highlighting its potential to predict life-threatening hemorrhage. Despite its strengths, the study has some limitations. The sample size was relatively small, which may affect the generalizability of the findings. Further research is needed to determine whether the implementation of this method in obstetric hemorrhage management bundles can lead to a significant reduction in severe PPH and maternal mortality in larger, more diverse populations.

In conclusion, this study presents a novel and practical approach to the early detection of severe PPH, leveraging both an innovative ex vivo model and a simple clinical maneuver. By providing a rapid, reliable, and non-invasive tool for identifying intractable PPH, this research represents a valuable contribution to maternal healthcare and underscores the importance of continued innovation and research in this critical field.

## Supplementary Information

Supplementary Information 1.

Supplementary Information 2.

Supplementary Information 3.

## Data Availability

Original video can be available from an optional access repository 10.5281/zenodo.13895692. The other data are available from the corresponding author on reasonable request.

## References

[1] Say, L. et al. Global causes of maternal death: A WHO systematic analysis. *Lancet Glob. Health* **2**, e323-333 (2014). 25103301 10.1016/S2214-109X(14)70227-X

[2] Escobar, M. F. et al. FIGO recommendations on the management of postpartum hemorrhage 2022. *Int. J. Gynaecol. Obstet.* **157**(Suppl 1), 3–50 (2022). 35297039 10.1002/ijgo.14116 PMC9313855

[3] Gallos, I. et al. Randomized trial of early detection and treatment of postpartum hemorrhage. *N. Engl. J. Med.* **389**, 11–21 (2023). 37158447 10.1056/NEJMoa2303966

[4] Akter, S. et al. Perceptions and experiences of the prevention, detection, and management of postpartum haemorrhage: A qualitative evidence synthesis. *Cochrane Database Syst. Rev.* **11**, CD013795 (2023). 38009552 10.1002/14651858.CD013795.pub2 PMC10680124

[5] Williams, E. V. et al. A cost-effectiveness analysis of early detection and bundled treatment of postpartum hemorrhage alongside the E-MOTIVE trial. *Nat. Med.* **30**, 2343–2348 (2024). 38844798 10.1038/s41591-024-03069-5 PMC11333277

[6] Kondoh, E. et al. CT scan assessment of intrauterine balloon tamponade failure for the treatment of atonic postpartum haemorrhage: Implications for treatment. *BJOG* **128**, 1726–1731 (2021). 33938132 10.1111/1471-0528.16724

[7] Kondoh, E., Chigusa, Y., Ueda, A., Mogami, H. & Mandai, M. Novel intrauterine balloon tamponade systems for postpartum hemorrhage. *Acta Obstet. Gynecol. Scand.* **98**, 1612–1617 (2019). 31339172 10.1111/aogs.13692

[8] Ikeda, A. et al. Novel subtype of atonic postpartum hemorrhage: Dynamic computed tomography evaluation of bleeding characteristics and the uterine cavity. *J. Matern. Fetal. Neonatal. Med.* **33**, 3286–3292 (2020). 30651015 10.1080/14767058.2019.1571033

[9] Hasegawa, J. et al. Current status of pregnancy-related maternal mortality in Japan: A report from the Maternal Death Exploratory Committee in Japan. *BMJ Open* **6**, e010304. 10.1136/bmjopen-2015-010304 (2016). 27000786 10.1136/bmjopen-2015-010304 PMC4809072

[10] Quantitative Blood Loss in Obstetric Hemorrhage. ACOG COMMITTEE OPINION, Number 794. *Obstet. Gynecol.* **134**, e150–e156 (2019). 31764759 10.1097/AOG.0000000000003564

[11] Hancock, A., Weeks, A. D. & Lavender, D. T. Is accurate and reliable blood loss estimation the “crucial step” in early detection of postpartum haemorrhage: An integrative review of the literature. *BMC Pregnancy Childbirth* **15**, 230. 10.1186/s12884-015-0653-6 (2015). 26415952 10.1186/s12884-015-0653-6 PMC4587838

[12] Koh, P. R., Di Filippo, D., Bisits, A. & Welsh, A. W. Bimanual examination for clot evacuation: a retrospective cohort study of women with postpartum haemorrhage after vaginal delivery. *BMC Pregnancy Childbirth* **20**, 245. 10.1186/s12884-020-02916-w (2020). 32334562 10.1186/s12884-020-02916-w PMC7183670

