# Supplementary material for: Early detection of intractable postpartum hemorrhage
Source: Sci Rep. 2025 Apr 3;15:11409. doi: 10.1038/s41598-025-96114-3 (PMC11968955; doi:10.1038/s41598-025-96114-3)
Supplement: Supplementary file 1 — Supplementary Information 1. [file 41598_2025_96114_MOESM1_ESM.pdf]

Figure S1

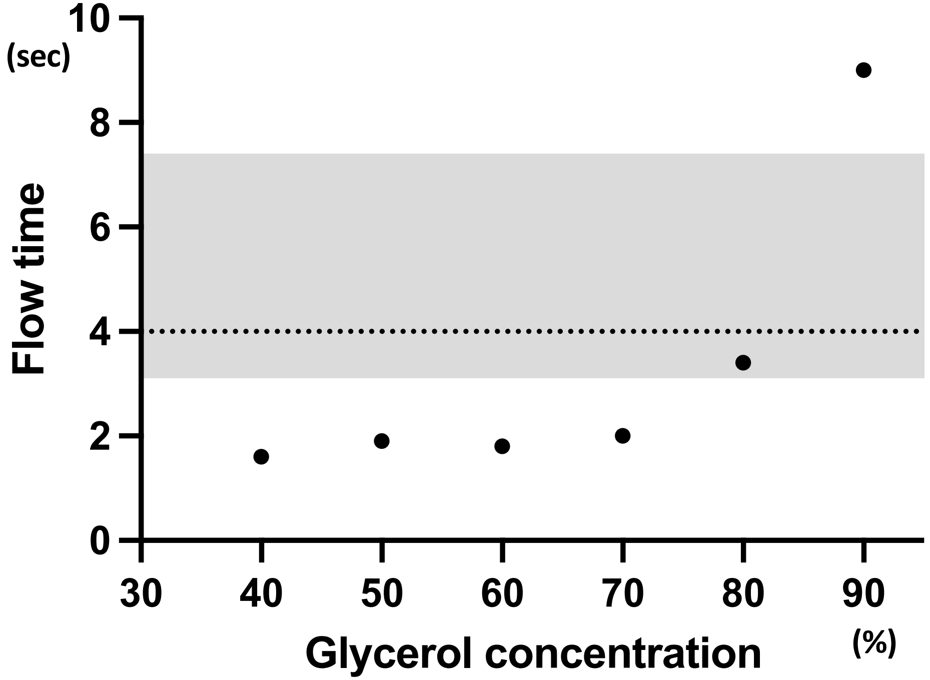

Figure S1. Glycerol solution concentration and flow time.

The flow rate decreases when the concentration of the glycerol solution exceeds 80%. The gray shading represents the range of times required for human blood to flow through the plastic container in seven cases. The dashed line indicates the median time for these cases.
