# Supplementary material for: Early detection of intractable postpartum hemorrhage
Source: Sci Rep. 2025 Apr 3;15:11409. doi: 10.1038/s41598-025-96114-3 (PMC11968955; doi:10.1038/s41598-025-96114-3)
Supplement: Supplementary file 2 — Supplementary Information 2. [file 41598_2025_96114_MOESM2_ESM.docx]

**Supplementary legend**

**Figure S1. Glycerol solution concentration and flow time.**

The flow rate decreases when the concentration of the glycerol solution exceeds 80%. The gray shading represents the range of times required for human blood to flow through the plastic container in seven cases. The dashed line indicates the median time for these cases.

**Table S1. Characteristics of patients transported due to postpartum hemorrhage.**

CD, cesarean delivery; CT, computed tomography; FFP, fresh frozen plasma; IBT, intrauterine balloon tamponade; PPH, postpartum hemorrhage; RBC, red blood cell; TI-V, time interval for bleeding to appear at the vagina; VD, vaginal delivery.

**Video S1. Simulation of PRACE using transparent uterine cavity model**
