# Supplementary material for: Early detection of intractable postpartum hemorrhage
Source: Sci Rep. 2025 Apr 3;15:11409. doi: 10.1038/s41598-025-96114-3 (PMC11968955; doi:10.1038/s41598-025-96114-3)
Supplement: Supplementary file 3 — Supplementary Information 3. [file 41598_2025_96114_MOESM3_ESM.pdf]

Table S1. Characteristics of patients transported due to postpartum hemorrhage.

Case no.	Age (years)	Gravity /parity	Gestational age at delivery (weeks)	Mode of deliverty	Primary /secondary PPH	Blood loss (mL)	Blood transfusion RBC/FFP (U)	Dynamic CT prior to hemostasis treatment	PRACE	Causes of PPH	Hemostatic technique		TI-V (sec)
											IBT with bilateral compression	Arterial embolization	
1	35	2/2	37	VD	Primary	6600	16/20	No	N/A	Suspected lower PRACE	Yes	No	0
2	28	4/3	39	VD	Primary	4900	18/12	No	N/A	Suspected lower PRACE	No	Yes	0
3	41	1/1	38	CD	Primary	8500	36/34	No	N/A	Placenta accreta spectrum	Yes	Yes	0
4	43	5/1	40	CD	Primary	8000	22/22	Yes	Yes	Upper PRACE	No	Yes	0
5	39	4/1	40	CD	Primary	8900	34/16	Yes	Yes	Lower PRACE	Yes	Yes	0
6	33	1/1	40	VD	Secondary	4600	12/16	Yes	Yes	Lower PRACE	Yes	No	1
7	36	3/3	40	VD	Primary	3800	18/14	No	N/A	Suspected lower PRACE	Yes	No	1
8	21	3/2	40	VD	Primary	2400	10/10	No	N/A	Suspected lower PRACE	Yes	No	1
9	31	3/3	37	CD	Primary	4500	12/14	No	N/A	Suspected lower PRACE	Yes	No	1
10	39	1/1	37	CD	Primary	9000	16/16	Yes	Yes	Lower PRACE	Yes	Yes	1
11	30	1/1	41	CD	Primary	4600	18/12	Yes	Yes	Lower PRACE	Yes	Yes	1
12	31	1/1	35	VD	Primary	2500	- / -	Yes	No	Retained placenta	No	No	5
13	29	1/1	40	VD	Secondary	2000	- / -	Yes	No	Unknown	No	No	6
14	29	2/2	38	CD	Primary	1400	- / -	Yes	No	Uterine atony	No	No	7
15	39	3/2	40	VD	Primary	2300	6/4	No	N/A	Uterine atony	No	No	8
16	35	3/3	40	VD	Primary	3300	12/10	Yes	No	Uterine atony	No	No	10
17	41	2/2	38	CD	Primary	4800	16/16	Yes	Yes	Lower PRACE	Yes	Yes	10
18	29	1/1	40	VD	Primary	2500	- / -	No	N/A	Uterine atony	No	No	10
19	37	2/1	34	CD	Primary	7000	14/12	Yes	No	Uterine atony	No	No	15
20	41	1/1	41	VD	Primary	2500	8/10	No	N/A	Uterine atony	No	No	15
21	27	1/1	41	CD	Primary	2300	6/ -	Yes	No	Uterine atony	No	No	15
22	48	4/1	36	CD	Secondary	4500	8/6	Yes	No	Uterine atony	No	No	15
23	38	2/1	41	VD	Primary	1500	- / -	Yes	No	Uterine atony	No	No	15
24	30	4/3	38	CD	Primary	1100	6/6	Yes	No	Uterine atony	No	No	15
25	40	6/3	40	VD	Primary	2100	- / -	No	N/A	Uterine atony	No	No	15
26	35	2/2	39	VD	Secondary	5100	6/12	Yes	No	Unknown	No	No	15
27	36	1/1	38	VD	Primary	4300	18/22	No	N/A	Retained placenta	No	No	15

CD, cesarean delivery; CT, computed tomography; FFP, fresh frozen plasma; IBT, intrauterine balloon tamponade; PPH, postpartum hemorrhage; RBC, red blood cell; TI-V, time interval for bleeding to appear at the vagina; VD, vaginal delivery.
